# Neuropharmacology of Ketamine and Its Use in the Treatment of Major Depressive Disorder: A Review

**DOI:** 10.7759/cureus.83244

**Published:** 2025-04-30

**Authors:** Zach Papadopoulos

**Affiliations:** 1 Department of Neuroscience, University of Pittsburgh, Pittsburgh, USA

**Keywords:** bdnf-trkb signaling, electroconvulsive therapy, glutamatergic neurotransmission, ketamine, major depressive disorder, neuropharmacology, nmda receptor antagonist, rapid-acting antidepressants, synaptic plasticity, treatment-resistant depression

## Abstract

Depression is a common yet potentially debilitating mood disorder with complex neurobiological underpinnings, including deficiencies in monoaminergic and glutamatergic signaling, overactivity of the lateral habenula, and dysregulation of brain-derived neurotrophic factor (BDNF) signaling. Ketamine has emerged as a mechanistically novel, effective, and rapidly acting antidepressant. Ketamine’s primary effects are due to N-methyl-D-aspartate receptor (NMDAR) antagonism, although hypotheses regarding the importance of its impact on monoaminergic signaling (preclinical evidence), BDNF signaling (preclinical evidence), opioid receptor agonism (preclinical evidence), and neuroinflammation (clinical evidence) have gained traction. Compared to selective serotonin reuptake inhibitors (SSRIs) and tricyclic antidepressants (TCAs), ketamine demonstrates greater efficacy, a significantly faster onset of action, and generally more tolerable side effects. However, its benefits are offset by a far shorter duration of antidepressant effects and accessibility limitations. In a head-to-head trial, compared to electroconvulsive therapy (ECT), ketamine showed similar efficacy in non-psychotic depression while providing clinically significant relief more rapidly. While promising, further research is needed to optimize ketamine’s dosing regimen, enhance its accessibility, and better understand potential drawbacks such as bladder toxicity and addiction potential. Additionally, studying the mechanisms behind ketamine’s antidepressant action may provide deeper insight into the neurobiology of depression.

## Introduction and background

The neurobiology of depression is complex and likely involves a multitude of different neurobiological mechanisms, including serotonergic, noradrenergic, and glutamatergic dysfunction [1]. Although depression is a common mood disorder, it can have severe consequences and is often implicated in suicide [2]. This paper will focus specifically on major depressive disorder (MDD), symptoms of which include sadness, fatigue, lack of interest in activities, hopelessness, and anhedonia [2]. 

The current first-line pharmacological treatments for depression are selective serotonin reuptake inhibitors (SSRIs), which target the serotonergic system. They are similar to their predecessors, tricyclic antidepressants (TCAs), which targeted both the serotonergic and noradrenergic systems. However, recent research implicates the glutamatergic system in addition to monoaminergic systems [1]. Moreover, SSRIs and TCAs have many drawbacks, such as limited efficacy, a slow speed of onset, and a multitude of side effects. Electroconvulsive therapy (ECT) remains the most effective treatment option for depression; however, its use is often limited by cognitive side effects, although modern techniques have sought to reduce these adverse effects [3]. These findings have driven the search for pharmacological interventions that more comprehensively address the complex neurobiological causes of depression without the drawbacks of traditional treatments. 

Ketamine is an anesthetic drug first synthesized to provide a safer alternative to phencyclidine, with fewer side effects and a shorter half-life [4]. Since 2000, growing interest has emerged in ketamine's potential as a novel antidepressant, given its unique pharmacological profile and rapid onset of action. Intravenous ketamine has demonstrated strong efficacy for depression in clinical trials and offers several advantages over TCAs and SSRIs. These qualities make ketamine one of the most significant breakthroughs in depression treatment over the past 50 years [1].

This review aims to offer a concise and accessible overview of recent literature on the neurobiology of depression and the use of ketamine as an antidepressant, particularly in treatment-resistant depression. This paper will explore (1) depression, its history, and its neurobiological underpinnings; (2) current treatment options for depression, focusing on their neuropharmacology and efficacy; and (3) ketamine, covering its origins as a dissociative anesthetic, its complex neuropharmacology, and its contemporary use for MDD [1]. This review will conclude with an analysis of how ketamine compares to other contemporary treatment options for depression. 

## Review

Methods

This review was conducted using a structured search of peer-reviewed literature on PubMed and Google Scholar published between 2005 and 2024. The search was performed on January 13, 2025. Search terms included combinations of “ketamine for depression,” “major depressive disorder,” “ketamine BDNF,” and “electroconvulsive therapy.” Boolean operators (AND, OR) were used to combine terms. Only English-language articles were considered. Inclusion criteria included primary research, meta-analyses, and review papers. Exclusion criteria included articles solely focused on psychiatric disorders other than depression, non-English publications, and abstracts without full text available. Articles were selected based on relevance to the neurobiology of depression, ketamine's mechanism of action, and clinical applications. Given the narrative nature of this review, no formal risk of bias assessment was performed, but an effort was made to include high-quality and recent studies where available. Data were synthesized to provide an overview of major themes in the existing literature. 

Depression: overview

Depression is the leading cause of disability worldwide and a potentially life-threatening mood disorder, especially if inadequately treated [2]. Not only is depression associated with high morbidity, but according to the World Health Organization, over 300 million people worldwide suffer from depression [2, 5]. Depression is the primary contributor to suicide, which is a prominent cause of death in teenagers and young adults as well as the elderly [2]. According to the American Psychiatric Association’s Diagnostic and Statistical Manual of Mental Disorders, fifth edition (DSM-5), depression encompasses several subtypes, including MDD, persistent depressive disorder, disruptive mood dysregulation disorder, and premenstrual dysphoric disorder [2]. This paper will focus predominantly on MDD. 

The main symptoms of MDD include a loss of interest, loss of hope in life, persistent feelings of sadness, and anhedonia [2]. Environmental factors, genetic predisposition, and neurobiological abnormalities are implicated in depression [2]. Studies indicate that genetics is responsible for between 30% and 40% of the risk of developing depression; moreover, the risk of developing depression increases if a first-degree relative is depressed, indicating a strong genetic component [2]. The remaining 60% to 70% of the risk of developing depression is attributed to environmental factors, such as stress, trauma, and gene-environment interactions [2]. Most relevant to this paper is the role that neurobiology plays in the pathophysiology of depression.

Depression: neurobiology

The monoamine hypothesis, which proposes that depression is caused by deficient levels of monoamines, such as norepinephrine (NE), dopamine (DA), and serotonin (5-HT), was once the prevailing theory among researchers studying the neurobiological basis of depression; however, it is now often considered incomplete [2]. Three primary observations contributed to researchers’ adoption of the monoamine hypothesis [2]. Firstly, when treating tuberculosis patients with iproniazid, a monoamine oxidase inhibitor (MAOI), patients became notably less depressed [2]. Secondly, administration of imipramine, a TCA originally developed as a potential antipsychotic, alleviated depressive symptoms and increased sociability [2,6]. Thirdly, reserpine, an antihypertensive drug that works by blocking the vesicular monoamine transporter and thus reducing monoaminergic signaling, was found to exacerbate depressive symptoms and cause drowsiness [2]. Therefore, it became clear to researchers that deficient monoaminergic signaling is strongly correlated with depressive symptoms. 

The precise neurobiological mechanisms that underpin the monoaminergic deficiencies seen in depressive pathology are not known; however, several hypotheses have been proposed [2]. One such hypothesis implicates decreased monoamine synthesis, which may be caused by genetic abnormalities or inadequate nutrition [2]. Another proposed mechanism implicates overactivity of monoamine oxidase, an enzyme responsible for breaking down neurotransmitters like serotonin, dopamine, and norepinephrine, in the depletion of synaptic monoamines and the subsequent reduction in monoaminergic signaling [2]. An increase in the efficiency of monoaminergic transporters, such as the serotonin, dopamine, and norepinephrine transporters (SERT, DAT, and NET, respectively), has also been proposed, as it leads to more rapid removal of monoamines from the synaptic cleft [2]. Additionally, postsynaptic changes, such as disturbances in the binding between monoamines and their receptors, have also been suggested [2]. Furthermore, the lateral habenula modulates reward and affective states and is a brain region implicated in the neurobiology of depression [1,7]. The lateral habenula serves as a gateway between the limbic forebrain and the monoaminergic brainstem nuclei [8]. Increased firing of the lateral habenula suppresses the activity of the dopaminergic ventral tegmental area and the serotonergic raphe nuclei [8,9]. Therefore, overactivity of the lateral habenula is associated with reduced monoaminergic activity, which in turn contributes to depressive symptoms [1,8].

While there is no question that monoaminergic signaling dysfunction is correlated with depression, current research suggests that other factors may be at play, specifically, disruption of neurotrophic factors and dysfunction in glutamatergic signaling [1]. Both of these factors are very relevant to the potential mechanisms underlying ketamine’s antidepressant effects [2]. The brain-derived neurotrophic factor (BDNF) protein helps support the growth, function, and survival of neurons in the brain and is heavily involved in neuroplasticity processes and synaptogenesis; reduced BDNF levels have also been correlated with hippocampal atrophy [2]. In fact, essentially all antidepressant drugs induce neuroplasticity, often by directly binding to tropomyosin receptor kinase B (TrkB), the receptor for BDNF, and allosterically potentiating BDNF binding [9]. Deficiencies in BDNF have been suggested to cause alterations in the brains of depressed individuals [2]. Increased stress, which is a major risk factor for depression, reduces BDNF levels, specifically in brain regions involved in the regulation of mood, like the prefrontal cortex (PFC) and hippocampus [2]. Moreover, genetic studies have shown that reduced BDNF expression, as well as deficits in glutamatergic and GABAergic signaling, are correlated with an increased risk of depression [2]. Many neurobiological mechanisms have been put forward as to how exactly dysfunction in these systems may contribute to depression: (1) upregulation of N-methyl-D-aspartate (NMDA) receptors in certain brain regions can cause neuronal death, (2) lower efficiency of glutamate reuptake via the glutamate transporter and deficiencies of GABAergic interneurons can cause overexcitation of neurons and subsequent neuronal death, and (3) glutamatergic signaling deficiencies may lower synaptic plasticity [2]. 

While many pharmacological treatments for depression increase monoaminergic and BDNF signaling, they fail to address the other potential neurobiological contributors to depression, such as deficiencies in glutamatergic signaling and overactivity of the lateral habenula [1,2].

Depression: treatments

There are several clinical treatment options for depression that this paper will cover: TCAs, SSRIs, ECT, and ketamine. As monoaminergic deficiencies are correlated with depressive pathology, antidepressant medications have historically sought to target the monoaminergic systems. The first-generation antidepressants were monoamine oxidase inhibitors (MAOIs), which inhibit the metabolism of synaptic monoamines, and TCAs, which inhibit the reuptake of both serotonin and norepinephrine [2]. Second-generation antidepressants include SSRIs, serotonin and norepinephrine reuptake inhibitors (SNRIs), and bupropion, a norepinephrine and dopamine reuptake inhibitor [3]. This paper will focus on TCAs and SSRIs.

TCAs

When it was discovered that MAOI use was accompanied by potentially dangerous food and drug interactions and their efficacy was called into question, TCAs replaced them as the first-line treatment option for depression [5,6]. The FDA approved imipramine, a TCA, in 1959, and TCAs remained the primary pharmacological treatment for depression for the next 30 years [5,6]. While effective, TCAs are often accompanied by notable side effects, likely attributable to their antagonism of adrenergic, histaminergic, and muscarinic receptors, such as dry eyes and mouth, urinary retention, memory impairments, dizziness, and confusion [6,10]. Moreover, TCAs have a narrow therapeutic index, meaning even moderate overdoses may result in fatal cardiac arrhythmias [6]. In addition, gradual dose titration at the start of TCA treatment is essential to enhance tolerability and, in turn, improve adherence [3]. Therefore, despite their efficacy, TCAs have fallen out of favor due to their high discontinuation rates.

SSRIs

In the 1980s, pharmaceutical companies sought antidepressants with similar efficacy to TCAs but lower side effects and broader therapeutic indexes, leading to the development of SSRIs [3]. SSRIs elicit antidepressant effects by inhibiting the SERT and thereby increasing synaptic levels of serotonin, potentiating serotonin neurotransmission. In contrast to TCAs, SSRIs have minimal affinity for adrenergic, histaminergic, or muscarinic receptors [6]. Therefore, SSRIs are better tolerated and much safer in overdose than TCAs, but still present quite a few challenges: their side effects can still be quite severe, they have a delayed onset of action, and their efficacy is limited [1,3].

Side Effects

The side effects of SSRIs, while less severe than TCAs, can be quite unpleasant and include sexual dysfunction, insomnia, and nausea [2]. Some studies have shown that around half of those who take SSRIs will experience some degree of sexual dysfunction, such as anorgasmia, which can lead to significant stress [3]. In addition, emotional blunting is a common complaint of those who take SSRIs, as is an increase in anxiety upon initiation of treatment, which is associated with exacerbation of suicidal ideation [3]. 

Delayed Onset of Action

A hallmark of SSRI treatment is their delayed onset of action, despite the fact that, unlike TCAs, SSRIs can be given at therapeutically effective doses immediately upon initiation of treatment [5]. SSRIs typically take several weeks to months to show clinically relevant effects, which makes them less useful for patients experiencing suicidality; in contrast, ketamine's rapid onset has been considered transformative for the management of acute suicidality [1,5]. Some researchers hypothesize that this delayed onset of action is due to the neuropsychological actions of SSRIs that work to ameliorate negative emotional biases characteristic of depression [5]. If true, this would explain why SSRIs take time to show effects, as changing emotional processing on an unconscious level would require time before a consciously apparent improvement in mood is realized [5].

*Limited Effic*acy

SSRIs have limited efficacy, with around half of patients prescribed SSRIs not responding adequately to treatment [5]. Moreover, some studies have shown SSRIs to be quite ineffective at reducing anhedonic symptoms, which are very common in depressed patients, especially in those with particularly severe and treatment-resistant depression (TRD) [5]. This limited efficacy, even compared to older antidepressants such as TCAs, may in part be due to SSRIs primary effect on the serotonergic system and lack of ability to increase synaptic levels of other neurotransmitters such as norepinephrine, dopamine, or glutamate [5].

ECT

ECT is a non-pharmacological antidepressant treatment that has been used for nearly a century [3]. It is the most effective option for TRD, defined as an inadequate response to two or more antidepressant medications [3,11]. ECT involves inducing controlled epileptic seizures by passing current through an electrode attached to the skull [3]. Molecular mechanisms underlying ECT’s efficacy as an antidepressant treatment remain unclear. Some studies indicate that the antidepressant effects of ECT may be due to upregulation of BDNF signaling, although results are mixed [12]. Furthermore, studies indicate a correlation between ECT and changes in monoaminergic signaling, including the downregulation of 5-HT2A and D2 receptors [13]. However, their precise role in ECT's antidepressant effects remains uncertain. Although ECT acts more quickly than first-line antidepressant treatments like SSRIs, multiple sessions are usually required to achieve clinically significant results [3]. Regarding the side effects of ECT, there is ongoing debate about the risk of permanent and severe cognitive deficits. While some research suggests that cognitive side effects are common but generally transient, clinicians often express far greater concern [11,14].

Ketamine: overview

Ketamine’s predecessor was phencyclidine (PCP), a very potent anesthetic first synthesized in 1956 [4]. Though effective as an anesthetic, its side effect profile, which includes salivation, hallucinations, prolonged emergence delirium following anesthesia, and dangerous increases in blood pressure, prompted researchers to seek alternative medications [1,7]. Ketamine was synthesized in 1962 as a shorter-acting, less hallucinogenic, and safer alternative to PCP and was approved by the FDA for use in anesthesia in 1970 [1,7]. When first given to patients, ketamine induced feelings of analgesia, floating, and dissociation from their bodies, which is why ketamine is now referred to as a “dissociative anesthetic” [4]. Owing to ketamine’s unique and complex pharmacology, it has proven effective not only for anesthesia and pain management but also for depression [4]. As a chiral arylcyclohexylamine, ketamine’s enantiomers both exhibit antidepressant effects, albeit to different extents [1,15]. Notably, (S)-ketamine has been approved as a nasal spray antidepressant in the United States and several European countries [1,15].

Ketamine: neuropharmacology

Understanding the neuropharmacology of ketamine is crucial in elucidating precisely how it elicits antidepressant effects. Researchers refer to ketamine as a "dirty" drug, meaning it affects many different receptor types in the brain, which may explain its wide range of clinical applications [4]. Unlike most other anesthetics, which primarily act as GABAA agonists, ketamine’s primary effects are due to its noncompetitive antagonism of N-methyl-D-aspartate receptors (NMDARs) [4]. In addition, ketamine targets GABA, opioid, serotonin, acetylcholine, and dopamine receptors, although in most cases with far weaker affinity than its affinity for NMDAR antagonism [1,6]. While ketamine’s primary effects are generally attributed to its NMDAR antagonism, there are several theories regarding the precise mechanisms by which ketamine produces antidepressant effects [4]. This paper will cover four of these theories: the NMDA receptor theory, the monoamine theory, the opioid receptor theory, and the anti-inflammatory theory [4]. However, ketamine’s antidepressant effects are likely multifaceted, with no one mechanism solely responsible. 

NMDAR Theory

**Figure 1:**
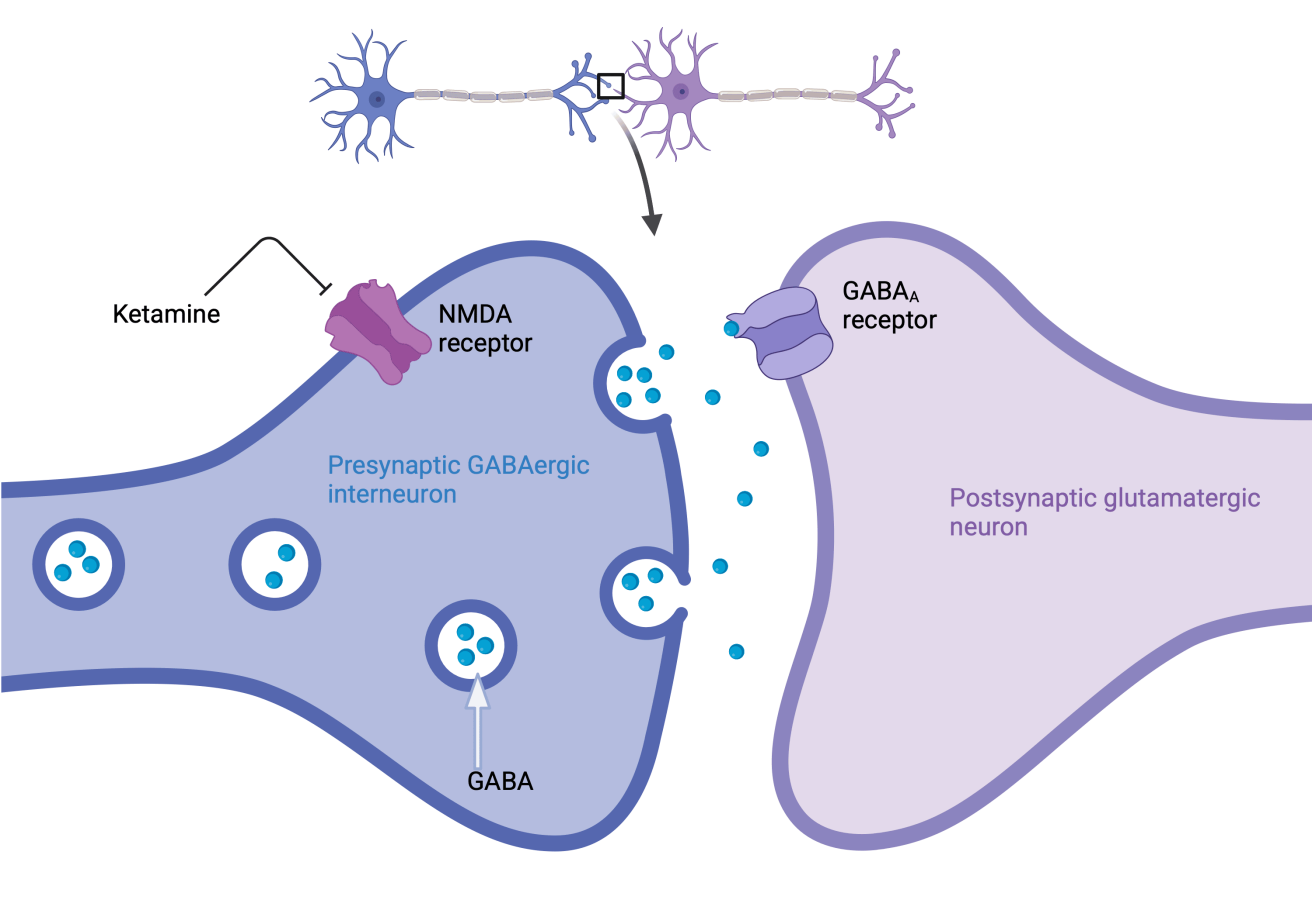
Proposed mechanism for ketamine’s antidepressant effects Ketamine inhibits NMDARs on GABAergic interneurons, leading to decreased inhibition of glutamatergic pyramidal neurons. Increased firing of these pyramidal neurons causes greater postsynaptic AMPAR activation, and ketamine antagonizes postsynaptic NMDARs. These actions both mediate an increase in BDNF-TrkB signaling, and thus an increase in neuroplasticity and antidepressant effects. This figure was created by the author. NMDARs: N-methyl-D-aspartate receptors; AMPAR: α-amino-3-hydroxy-5-methyl-4-isoxazolepropionic acid receptors; BDNF: brain-derived neurotrophic factor; TrkB: tropomyosin receptor kinase B

**Figure 2:**
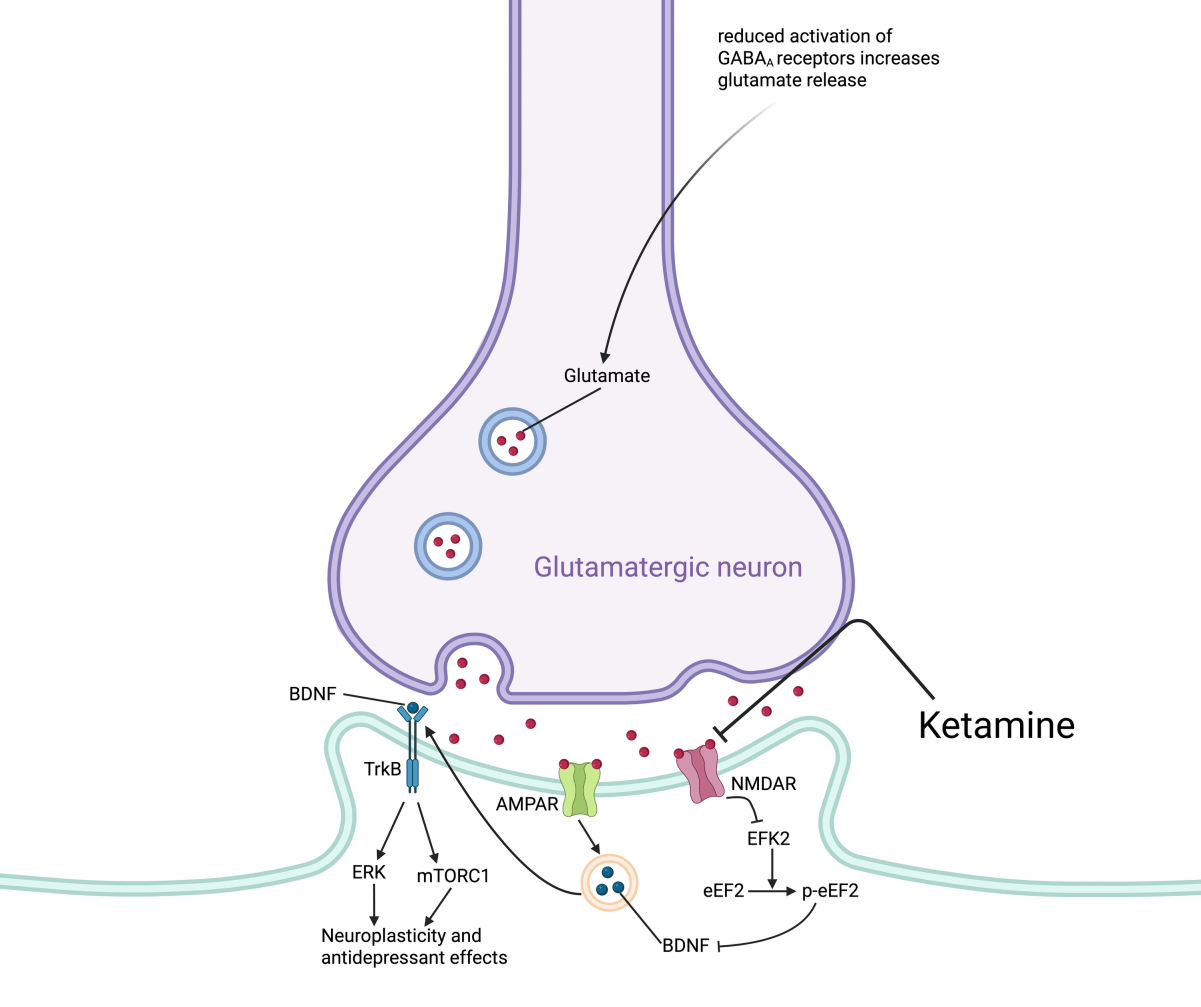
Proposed mechanism for ketamine’s antidepressant effects. Ketamine inhibits NMDARs on GABAergic interneurons, leading to decreased inhibition of glutamatergic pyramidal neurons. Increased firing of these pyramidal neurons causes greater postsynaptic AMPAR activation, and ketamine antagonizes postsynaptic NMDARs. These actions both mediate an increase in BDNF-TrkB signaling, and thus an increase in neuroplasticity and antidepressant effects. This figure was created by the author. NMDARs: N-methyl-D-aspartate receptors; AMPAR: α-amino-3-hydroxy-5-methyl-4-isoxazolepropionic acid receptors; BDNF: brain-derived neurotrophic factor; TrkB: tropomyosin receptor kinase B; EFK2: eukaryotic elongation factor-2 kinase; eEF2: eukaryotic elongation factor 2; ERK: extracellular signal-regulated kinase; mTORC1: mammalian target of rapamycin complex 1

The NMDAR theory is heavily supported and attributes ketamine’s antidepressant effects in part to its antagonism of NMDARs on presynaptic GABAergic interneurons that ordinarily inhibit glutamate release from glutamatergic pyramidal neurons [1]. This glutamate release activates postsynaptic α-amino-3-hydroxy-5-methyl-4-isoxazolepropionic acid receptors (AMPARs), which then leads to activation of synaptogenesis and neuroplasticity pathways [1]. The necessity of AMPAR activation for ketamine’s antidepressant effects is supported by preclinical work showing loss of ketamine’s antidepressant effects following administration of AMPAR antagonists [1,16]. There are several intracellular neuroplasticity pathways implicated in ketamine’s antidepressant action [1]. The aforementioned AMPARs downstream of the NMDARs antagonized by ketamine are responsible for activation of BDNF-TrkB signaling, which has already been implicated both in the neurobiology of depression and the neuropharmacological mechanisms behind existing depression treatments, as discussed earlier [1]. When BDNF binds to the TrkB receptor, both mammalian target of rapamycin complex 1 (mTORC1) and extracellular signal-regulated kinase (ERK) pathways are activated, which both result in increased translation of proteins required for synaptogenesis [1,17]. Ketamine’s antagonism of NMDARs causes suppression of eukaryotic elongation factor-2 kinase (EFK2), which catalyzes the reaction of eukaryotic elongation factor 2 (eEF2) to phosphorylated eEF2, ultimately relieving suppression of BDNF translation and enhancing neuroplasticity [1,4]. Research in animals has shown that upregulation of the BDNF and mTORC1 signaling pathways is necessary for ketamine to elicit antidepressant effects, lending credence to this proposed neurobiological mechanism underpinning ketamine’s antidepressant actions (Figures 1, 2) [1,4,17]. 

Monoamine Theory

While ketamine’s primary effects are largely attributed to its NMDAR antagonism, the monoamine theory proposes that ketamine’s antidepressant effects partially stem from its effects on monoaminergic signaling, specifically via inhibition of reuptake of monoamines, reminiscent of the mechanisms of action of SSRIs and TCAs [4]. Preclinical studies indicate that ketamine inhibits SERT function, increasing synaptic serotonin levels, and that depletion of serotonin inhibits ketamine’s antidepressant effects, highlighting serotonin’s essential role in ketamine’s antidepressant action [1,4,18]. Animal studies suggest increased serotonin release in the medial PFC via AMPAR activation in the dorsal raphe nucleus, leading to increased mTORC1 signaling, and thus increased synaptogenesis, may be another way ketamine elicits antidepressant effects [1,19]. The potential role of dopamine in ketamine’s antidepressant action remains unclear; however, one study in mice found that activation of pyramidal cells in the medial PFC that express a specific dopamine receptor subclass was sufficient to induce antidepressant effects following ketamine administration [1,20]. Furthermore, blocking these receptors blocked ketamine’s antidepressant effects, suggesting a potential role for dopamine in ketamine’s antidepressant action [20]. However, the effects on monoaminergic signaling alone cannot fully explain the mechanisms behind ketamine’s antidepressant effects, as SSRIs and TCAs also modulate monoaminergic signaling but produce effects much more slowly [4]. 

Opioid Receptor Theory

Ketamine also acts on opioid receptors, primarily μ, κ, and, to a lesser extent, δ, forming the basis of the opioid receptor theory [1]. This theory is not as well established as that of the NMDA receptor or monoamine theories, with many studies coming to conflicting conclusions [1]. Some studies suggest that at anesthetic doses, ketamine interacts with μ and κ receptors but as an antagonist and does not interact with them at all at subanesthetic doses [4]. However, other studies suggest that ketamine may stimulate the release of β-endorphins, which can agonize μ and κ receptors, leading to BDNF upregulation and subsequent synaptogenesis, a process that, as discussed earlier, is strongly implicated in antidepressant effects [4]. Casting doubt on the opioid receptor hypothesis is an animal study that failed to show attenuation of ketamine’s antidepressant effects after administration of naltrexone, a μ receptor antagonist [1,4,21]. On the contrary, another animal study found that opioid receptor antagonists abolished ketamine’s ability to reduce overactivity in the lateral habenula, thereby abolishing its antidepressant effects [1,4,22]. Morphine, a μ receptor agonist, did not show similar antidepressant effects to ketamine, indicating that while opioid receptor agonism may be necessary for ketamine’s antidepressant effects, ketamine’s antidepressant action cannot be fully explained by μ receptor agonism [1]. 

Anti-inflammatory Theory

The anti-inflammatory theory of ketamine’s antidepressant effects is less researched than the other mechanisms explored in this paper, but is nevertheless important to mention. It is well established that depression is correlated with neuroinflammation, and ketamine has anti-inflammatory effects, both systemically and within the nervous system [4]. This is supported by a study that showed that ketamine decreased postoperative delirium after cardiac surgery,y that was caused by neuroinflammation [4,23]. Therefore, it is plausible that a reduction in neuroinflammation may contribute to ketamine’s antidepressant actions.

Ketamine: drawbacks

While ketamine shows significant promise as a novel, rapidly acting antidepressant, there are several noteworthy drawbacks: the side effects, abuse potential, and transience of the antidepressant effects [1]. 

Ketamine: side effects

Side effects of ketamine administration include, but are not limited to, dissociation, hallucinations, increased blood and intracranial pressure, nausea, headaches, blurred vision, dizziness, drowsiness, perceptual disturbance, and anxiety [1,4,24]. Blood pressure must be assessed both before and after ketamine treatment to ensure patients do not become dangerously hypertensive; continuous monitoring during infusion is also recommended by clinical guidelines [1]. A recent study involving 84 participants examined the physiological effects of subanesthetic doses of ketamine for depression and found mean blood pressure increases of 19.6 mmHg systolic and 13.4 mmHg diastolic, with 12% of participants requiring antihypertensive medication [25]. However, another study involving 66 patients and 0.5 mg/kg ketamine intravenous infusions administered over 40 minutes, which is typically how ketamine is administered for depression, found clinically insignificant mean blood pressure increases of 3.2 mmHg systolic and diastolic [25]. Moreover, the modest increases in blood pressure faded 70 minutes after the beginning of ketamine administration [25]. Therefore, while research clearly shows that ketamine administration for depression increases blood pressure, it remains unclear whether this increase is clinically significant. 

In addition, dissociative side effects are extremely common following ketamine administration, even after subanesthetic doses, and are often accompanied by other psychedelic-like effects such as synesthesia, derealization, depersonalization, and hallucinations [1]. A study of 188 participants who received subanesthetic doses of ketamine found that 78% described feeling “strange, weird, or bizarre”; 74% felt spacey; 72% felt woozy/loopy; 62% experienced dissociation; 57% reported visual distortions; 55% felt like they were floating; and 53% reported numbness [24]. Cognitive side effects may also be causally related to ketamine administration, with impairments in working memory and encoding of information into episodic memory being commonly reported; however, findings are mixed [1]. It is worth noting that the side effects covered in this section were largely transient, subsiding several hours after administration of ketamine [24]. 

There are also concerns regarding ketamine-induced urinary tract dysfunction, although these issues are most commonly reported in chronic and daily recreational users, as well as those given repeated high doses for pain [1,5]. Recreational users consume significantly higher doses of ketamine than are administered in clinical settings for depression; however, the FDA found that individuals using intranasal ketamine for depression exhibited a higher prevalence of urinary tract issues [1]. There is a small but growing number of case reports about urinary tract issues associated with ketamine use; however, the mechanism behind these adverse effects is unclear [26].

Ketamine: abuse potential

Ketamine’s potential for abuse is a concern among researchers given its widespread recreational use. Recreational users typically snort ketamine in powdered form, but some opt to inject it intravenously or intramuscularly [1]. There is no question that ketamine may lead to tolerance and addiction when used in large and frequent doses; however, whether or not there is a clinically significant risk of tolerance and addiction when administered for depression remains in question [1].

The precise mechanisms underpinning ketamine’s addictive properties have yet to be fully elucidated. However, researchers hypothesize that pleasant sensations or relief from negative feelings due to its dissociative effects, modulation of dopaminergic signaling, opioid receptor activation, and reward circuitry activation may all contribute [1]. One study found that mood improvements following ketamine administration correlated with increased activity in mesolimbic areas involved in reward processing, such as the orbitofrontal cortex, medial substantia nigra, ventral tegmental area (VTA), and nucleus accumbens (NAcc) [27]. Given that increased DA release in the NAcc via the mesolimbic pathway is highly implicated in the neurobiology of addiction, this finding suggests a potential link between ketamine’s antidepressant and addictive properties. Furthermore, individuals with MDD often show increased activity in specific mesolimbic regions, such as the amygdala and insula, in response to negative stimuli [27]. Research indicates that ketamine reduces this hyperactivity, potentially reinforcing its use by alleviating the psychological impact of negative stimuli. These findings provide a plausible neurobiological mechanism underlying ketamine’s addictive potential: by simultaneously increasing activity in certain mesolimbic structures and dampening the overreactivity of others in response to negative stimuli, ketamine may promote addiction [27]. Whether long-term ketamine use for depression results in tolerance and subsequent withdrawal symptoms upon discontinuation remains unclear. Recreational users occasionally describe withdrawal symptoms, such as palpitations, anxiety, shaking, and sweating, upon cessation of use; however, concrete evidence of a ketamine-induced withdrawal syndrome is lacking [1]. Notably, a study of 297 participants who received ketamine for depression found no evidence of a ketamine-induced withdrawal syndrome [28]. 

Ketamine: transience of antidepressant effects

While ketamine’s ability to very rapidly and consistently produce antidepressant effects is noteworthy, these effects are transient, often dissipating within a week or two after treatment, limiting its clinical utility [1]. Some research indicates that repeated ketamine infusions rather than one single administration can help marginally prolong its antidepressant effects, even after treatment is stopped [29]. Interestingly enough, one study found that twice-weekly infusions were just as effective as thrice-weekly infusions, suggesting that ketamine infusions more than twice a week may be unnecessary [30]. The optimal frequency of ketamine infusions remains unclear; however, multiple infusions consistently show longer-lasting antidepressant effects relative to a single infusion, and ongoing clinical trials are investigating the role of maintenance infusions. 

Ketamine: comparison to other treatments

Common conventional treatments for depression include antidepressant medications, such as SSRIs and TCAs, as well as ECT [2]. Ketamine differs quite markedly from these conventional treatments in five important areas: response rates, speed of onset, duration of action, side effects, and accessibility. 

Response Rates

There are notable disparities in the efficacy of SSRIs, TCAs, ECT, and ketamine. ECT has been the most effective antidepressant treatment for nearly 80 years, prompting researchers to seek pharmacological treatment options for depression that offer non-inferior efficacy. SSRIs and TCAs generally exhibit similar efficacy in treating depression, though TCAs may be marginally more effective, likely due to their dual noradrenergic and serotonergic action [3]. In comparison, ketamine is efficacious in a greater proportion of patients than SSRIs and TCAs. SSRIs and TCAs often show response rates of under 50%, while most studies show 50-70% efficacy rates for ketamine, which is similar to ECT [1,5,15]. One randomized controlled trial in Europe in patients with depression, including those with psychotic symptoms, showed that while ketamine was an effective antidepressant, it was inferior to ECT in terms of both reduction of depressive symptoms and the rates of remission [3]. However, a later randomized controlled trial that excluded participants with psychotic symptoms found that an antidepressant response, measured by the Quick Inventory of Depressive Symptomatology-Self-Report 16, occurred in 55.4% of patients receiving ketamine versus 41.2% receiving ECT, indicating noninferiority of ketamine as compared to ECT (difference, 14.2 percentage points; 95% confidence interval, 3.9 to 24.2; p < 0.001 for the noninferiority of ketamine to ECT) [11]. The discrepancy between the results of these two studies may be explained by the fact that ECT is particularly effective for individuals with psychotic depression [3]. These findings are noteworthy, as they suggest that a pharmacological treatment may achieve efficacy comparable to ECT [3]. The discrepancy in the results of these two studies may be explained by the fact that ECT is particularly effective for individuals with psychotic depression [3]. The results are surprising, as they suggest a pharmacological treatment may be as effective as ECT [3].

Speed of Onset 

There are quite drastic differences between ketamine and other pharmacological treatments for depression when it comes to the speed of onset. Compared to SSRIs and TCAs, which take several weeks to show effects, ketamine is able to elicit antidepressant effects within hours that increase over the following days [1,3,15]. ECT is known for its effectiveness and rapid action as an antidepressant treatment, working far more rapidly than SSRIs and TCAs [3]. However, it typically requires multiple sessions before clinically significant antidepressant effects are achieved [3]. Therefore, ketamine is superior to these treatments in terms of speed of onset, which proves especially important in cases of suicidality.

Duration of Effects

The duration of antidepressant effects after cessation of treatment varies between these therapies. Ketamine’s antidepressant effects typically dissipate within a week or two after the final dose [1]. In contrast, a meta-analysis of 40 randomized controlled trials found that, compared to placebo, TCAs and SSRIs both showed reduced relapse rates six months after remission from depression and discontinuation of treatment, with TCAs demonstrating moderate superiority over SSRIs [31]. This indicates that TCAs and SSRIs are able to elicit antidepressant effects months after cessation of use [31]. In contrast, ketamine and ECT show similar times to relapse, both of which are inferior to TCAs and SSRIs. Therefore, TCAs provide the longest-lasting antidepressant effects, followed by SSRIs, while ketamine and ECT exhibit the shortest duration of efficacy [4,11]. 

Side Effects

The side effects of these treatments vary. SSRIs commonly cause sexual dysfunction, insomnia, and nausea [2]. TCAs have a higher side effect burden than SSRIs, with additional risks including cardiac toxicity, orthostatic hypotension, cognitive deficits, and cholinergic side effects, alongside the symptoms seen with SSRIs [3]. As for ECT, while headaches and cognitive side effects are highly prevalent following treatment, research suggests they are typically transient; however, clinicians tend to be less optimistic about the time course of side-effect resolution [3]. Ketamine administration can cause dissociation, hallucinations, blood pressure increases, and headaches, among other things; however, these side effects typically fade within a few hours after treatment [24]. Overall, ketamine is well tolerated relative to other treatments, especially TCAs.

Accessibility

Accessibility to these treatments affects their clinical utility. SSRIs, as the first-line pharmacological treatment option for depression, and TCAs are both widespread and affordable treatment options with costs typically reaching a few hundred dollars per year [5,32]. They are taken orally and are generally covered by insurance and available at most pharmacies. On the contrary, ketamine and ECT face accessibility challenges. Ketamine for depression is limited by cost and accessibility. It is available in both intravenous and intranasal formulations, with the intranasal formulation proving more convenient as it is able to be administered at home without the aid of a healthcare professional [1,33]. The intravenous formulation can cost upwards of $1000 per session, while the intranasal formulation is even more expensive [34]. As for ECT, the need for general anesthesia and a facility to perform the procedure inherently makes it less accessible than other treatments [11]. Moreover, ECT typically costs upwards of $1000 per session and $10,000 per year [34]. Overall, SSRIs and TCAs are much more accessible than ketamine or ECT. A comparison of ketamine, SSRIs, TCAs, and ECT in the treatment of MDD is presented in Table 1.

**Table 1:** Comparison of ketamine, SSRIs, TCAs, and ECT in the treatment of major depressive disorder SSRIs: selective serotonin reuptake inhibitors; TCAs: tricyclic antidepressants; ECT: electroconvulsive therapy

Treatment	Onset of action	Response rate	Duration of effects	Tolerability	Accessibility
Ketamine	Hours	50%–70%	Days to weeks	Transient dissociation and hypertension	Upwards of $1000 per treatment for IV
SSRIs	Several weeks	40%–50%	Months	Sexual dysfunction, weight gain, insomnia, nausea	Widespread and affordable
TCAs	Several weeks	40%–50%	Months	Antimuscarinic side effects, cardiotoxicity, plus typical SSRI side effects	Widespread (but prescribed less today) and affordable
ECT	After several treatment sessions	50%–70%	Similar to ketamine	Potentially permanent cognitive side effects	Upwards of $1000 per session

Limitations

This article is a narrative review and does not follow a systematic methodology for study selection, which introduces potential selection and publication bias. No meta-analysis was performed, and the findings are based on a synthesis of key studies deemed relevant by the author. Therefore, some potentially important studies may not have been included, and the results should be interpreted with appropriate caution. 

A narrative review was chosen for this paper because the existing literature on the neuropharmacology of ketamine and its use in the treatment of major depressive disorder is heterogeneous and evolving, making systematic methodologies impractical. The narrative review format allowed for a broad synthesis of the results of preclinical studies, clinical trials, and reviews relevant to the topic. 

## Conclusions

Ketamine represents a major breakthrough in the treatment of depression, offering a rapid onset of action, high response rates, and a novel mechanism of action relative to traditional antidepressant treatments. While ketamine offers similar efficacy in non-psychotic depression compared to ECT with fewer permanent cognitive side effects, further research is needed to clarify its long-term safety and optimal use across different depression subtypes. Ketamine's clinical utility is hindered by its short duration of antidepressant effects and accessibility challenges. Clinically, ketamine should be considered in patients with TRD, particularly those with suicidality, recognizing its rapid onset and emerging but still incomplete long-term safety data. 
